# Methods for smoking cessation and treatment of nicotine dependence

**DOI:** 10.1016/S1808-8694(15)31254-4

**Published:** 2015-10-20

**Authors:** Aracy Pereira Silveira Balbani, Jair Cortez Montovani

**Affiliations:** Ph.D. in Medicine, Volunteer Professor, Discipline of Otorhinolaryngology and Head and Neck Surgery, Medical School, Botucatu, Universidade Estadual Paulista “Júlio de Mesquita Filho” (UNESP); Full Professor, Discipline of Otorhinolaryngology and Head and Neck Surgery, Medical School, Botucatu, Universidade Estadual Paulista “Júlio de Mesquita Filho” (UNESP)

**Keywords:** smoking, nicotine, tobacco use disorder, tobacco use cessation, bupropion

## Abstract

Smoking is related to 30% of cancer deaths. It is a risk factor for respiratory tract, esophagus, stomach, pancreas, uterine cervix, kidney and bladder carcinomas. Nicotine induces tolerance and addiction by acting on the central dopaminergic pathways, thus leading to pleasure and reward sensations within the limbic system. It stimulates the central nervous system (CNS), enhances alertness and reduces the appetite. A 50% reduction of nicotine consumption may trigger withdrawal symptoms in addicted individuals: anxiety, anger, sleep disorders, hunger, cognitive dysfunction and cigarette craving. Medical advice is the cornerstone of smoking cessation. Pharmacotherapy of nicotine addiction comprises first-line (bupropion and nicotine replacement therapy) and second-line (clonidine and nortriptyline) drugs. Bupropion is a non-tricyclic antidepressant that inhibits dopamine uptake, whose contraindications are: epilepsy, eating disorders, uncontrolled hypertension, recent alcohol abstinence and current therapy with MAO inhibitors. Nicotine replacement therapy can be done with patches or gums. Counseling groups and behavioral interventions are efficacious. The effects of acupuncture on smoking cessation are not fully elucidated. Prompt smoking cessation or gradual reduction strategies have similar success rates.

## INTRODUCTION

Tobacco is produced by two plant species - Nicotiana tabacum and Nicotiana rustica, which come from the Peruvian and equatorial Andes. These plants were found out approximately 18,000 years ago, when Asiatic populations migrated to America^1^.

When Christopher Columbus arrived in the New World, the cultivation and use of tobacco had already been disseminated among Indians over the continent. It had several applications, including religious rituals and as insecticide in agriculture. Tobacco was smoked in pipes, inhaled, chewed, eaten, and drunk as tea. Important medicinal plant, it was used for intestinal wash-ups, skin smear to kill louses, instilled as eye-wash and used in ointments, analgesic and antiseptic formulations^1^.

Aware of smoking habits and the tobacco medicinal properties, explorers decided to take the plant seeds to Europe. In Portugal and in Spain, tobacco was cultivated in royal palace gardens, while nobles used it to fight cancer. Rapidly, tobacco became much valuable in Europe, while English pirates invaded and plundered Spanish ships coming from America, till the British government decided to cultivate the plant in several colonies^1^.

In 1850, the first manufactured cigarettes were sold in England, whose consumption became popular during the First World War. Smoking habits summit took place in the 50 and 60's, declining in certain countries from 1970 and on^1^. Today, there are more than a billion tobacco users around the world, among which 90% started smoking in the adolescence^2^.

From 1920's, the increase of lung cancer incidence was observed, which was confirmed in several studies thirty years later. In 1971, a formal report was published in the United States confirming that “smoking negatively affects human health and contributes for the onset of severe diseases”^1^.

Currently, the World Health Organization accounts for more than four millions fatal victims caused by cigarette each year^2^. It is known that smoking is related to at least 30% of cancer deaths. It is a risk factor for the onset of lung, mouth, pharynx, larynx, esophagus, stomach, pancreas, uterine cervix, kidney and bladder carcinomas^3^. Moreover, morbidity by cardiovascular and cerebrovascular diseases, chronic obstructive pulmonary diseases (COPD) and peptic diseases, as well as other affections, is higher among smokers^1^.

In 1988, a new North-American report concluded that nicotine found in cigarettes and in other tobacco products is a drug that causes dependence. It is estimated that 24% of the adult population in several countries, Brazil included, are nicotine dependent^4^.

Frequently, otorhinolaryngologists receive smokers with inflammatory or tumoral diseases of the upper airways. It is fundamental to diagnose and treat the chemical dependence in these patients, promoting smoking cessation in order to prevent or cure these affections.

The authors present a pharmacology outlook, addressing actions and nicotine dependence, as well as treatment modalities available for smoking cessation which may be prescribed by otorhinolaryngologists.

###  

#### Literature Review

Indexed studies were reviewed through Lilacs and Medline databases under the keywords in Portuguese, such as “tabagismo”, “nicotina”, “transtorno por uso do tabaco”, “abandono do uso do tabaco”, “bupropiona” or its related links in English: “smoking”, “nicotine”, “tobacco use disorder”, “tobacco use cessation”, “bupropion”.

#### Nicotine Pharmacology

Cigarette smoke consists of volatile chemical substances (92%) and particulate material (8%) resultant of tobacco combustion^5^. Nicotine, a tertiary volatile amine, is the most important active tobacco component^4^^,^^6^. When tobacco coal's temperature reaches 800°C, racemic shapes of nicotine emerge, which form four nitrosamines with cancer potential^7^. However, nearly 35% of nicotine is destroyed during cigarette combustion; more than 35% is lost in non-inhaled smoke and 8% is not smoked^6^. Therefore, each cigarette contains 7–9 mg of nicotine, of which a little more than 1 mg is absorbed by the smoker^4^. Cured tobacco's nicotine for pipes and cigars is alkaline and it is more easily absorbed through the mouth. On the other hand, cigarette's nicotine is acid, therefore it is practically not absorbed by the mouth mucosa, and has to be inhaled to be absorbed by the lungs^1^^,^^7^.

Nicotine is rapidly absorbed by the lung alveolus and reaches the brain within 10 seconds. Its half-life is of approximately 2 hours, and metabolization is mostly hepatic, through P450 cytochrome. The main enzyme involved is CYP2A6. Molecular biology studies demonstrate that capacity in metabolizing nicotine varies according to each individual7. CYP2A6*2 and CYP2A6*3-allele individuals are less prone to be smokers, and if they do, they tend to consume less tobacco than CYP2A6*1-allele individuals^8^.

Vasconcelos et al. (2005)^9^ analyzed the genetic profile of CYP2A6 in a sample of an adult Brazilian population, composed by 147 Caucasian individuals, 142 Mulattos and 123 Blacks, among which 205 were smokers or ex-smokers, and 207 were non-smokers. The alleles mostly found in this sample were: CYP2A6*1B (29.9%), CYP2A6*2 (1.7%), CYP2A6*4 (0.5%) and CYP2A6*9 (5.7%). Contrary to expectations, frequency of CYP2A6*1B-allel individuals among non-smokers was higher. Distribution of CYP2A6*1B alleles also presented racial differences, with decreasing frequency among Caucasians, Mulattos and Blacks. Presence of this allele was associated with a higher probability of nicotine dependency among Caucasians (a 14-fold higher risk) and Mulatto (a 3-fold higher risk), but not among Blacks.

The most important nicotine metabolite is cotinine, which can be detected in urine, saliva and blood^7^. Only 5% of nicotine is excreted without alterations by the kidneys^4^.

#### Nicotine actions

The systemic actions of nicotine are mediated by nicotinic receptors found in the central nervous system (CNS), peripheral autonomic nodes, supra-renal glands, sensitive nerves and the skeletal striated muscle^4^.

Nicotine's main acute effects over the cardiovascular system are^10^: peripheral vasoconstriction, increase of the blood pressure and heart rate. Nicotine also interferes in the endocrine system, yielding the release of antidiuretic hormone and water retention. In the gastrointestinal system, nicotine acts parasympathetically, stimulating tonus increase and intestinal motor activity^10^.

In nervous endings, nicotine stimulates release of the following neurotransmitters: acetylcholine, dopamine (DA), glutamate, serotonin and gamma aminobutyric acid (GABA)^11^.

Nicotine is CNS stimulant, leading to increased alertness and to reduced appetite. After a draft, the sensation may be compared to that described by amphetamine, heroine, cocaine and crack users^4^. The main sensations may include dizziness, nauseas and vomiting^10^.

Rose et al. (2003)^11^ studied nicotine's acute effects over the brain blood flow in adults through tomography by positrons emission (PET). Nicotine interferes in the reticular formation blood flow, including areas of the pons, mesencephalus and thalamus, and plays a role in awareness and awakening mechanisms. Low doses of nicotine have a central stimulating effect, while higher doses have a depressing effect. Nicotine also leads to dose-dependent increase of blood flow in the left hemisphere amygdala, which may explain the anxiolytic effect of smoking.

Experimental studies show that nicotine acts as enzymatic inductor in the liver. This way it reduces half-life of several medicines such as: local anesthetics, morphine, codeine, teophyline, heparin, warfarin, amitriptyline, imipramine, propranolol, chlorpromazine, diazepam, chlordiazepoxide and indometacin. Thus, smokers may require larger doses of these medicines to have the expected therapeutic effects^10^.

Ingestion of nicotine-based insecticides may cause acute intoxication, with the following symptoms: salivation, vomiting, muscle weakness, prostration, cold sudoresis, mental confusion and hypotension. In severe cases (ingestion of over 60 mg of nicotine), chronic convulsions and respiratory failure may occur^10^.

#### Nicotine Chemical Dependency

Nicotine induces tolerance (need of progressively higher doses to obtain the same effect) and dependence (desire of consumption) as it acts in the dopaminergic pathways of the mesolimbic system, reducing the thalamus activity^4^. Similarly to other psychoactive drugs, it releases dopamine in the nucleus accumbens, located in the mesencephalus, stimulating a pleasant and “rewarding” sensation^8^^,^^12^. After the discomfort caused by the first drafts of tobacco (sickness, dizziness, nausea), the smoker experience a pleasant sensation with the use of nicotine^7^.

According to Marques et al. (2001)^4^, a 50% decrease in nicotine consumption is enough to trigger withdrawal symptoms in dependent individuals. Nicotine abstinence syndrome is mediated by noradrenalin and starts 8 hours after the last cigarette, reaching a peak on the third day. Main symptoms include: anxiety, irritability, sleep disorders (insomnia and daytime sleepiness), appetite increase, cognitive disorders (decrease of concentration and attention) and craving. That is why nicotine-dependent individuals present abstinence relief when they smoke their first morning cigarette.

Irritability during nicotine withdrawal is a common smokers' complaint^4^. Our experience includes a 34-year-old patient that used to smoke about 12 cigarettes per day and had quit smoking for two months. The patient says she had physically improved during withdrawal, but started smoking again due to her husband's insistence: “He could no longer stand my bad mood”.

Unwanted weight gain is one of the symptoms that mostly upset patients under nicotine abstinence. Mostly, weight gain gets around 4 to 6 Kg^13^, and in some people it may reach 10% of body weight^4^.

Women and smokers that smoke over 25 cigarettes per day tend to gain weight after smoking cessation, probably due to food ingestion and metabolic adjustments^4^.

Epidemiologic studies show that more than 70% of smokers want to quit smoking^8^. However, less than 10% reach their goal by their own, as discomfort caused by nicotine abstinence and craving leads most ex-smokers to relapse^8^^,^^13^. Relapses usually occur between two days and three months of withdrawal^13^.

Cox et al. (2003)^3^ alert that 58% of cancer patients continue smoking after diagnosis, usually due to behavioral habit, anxiety or stress.

#### Treatment of Nicotine Dependency

Medical support may enhance the success rate in smoking cessation^13^.

#### Patient and Family Counseling

Talking with the patient is the first step for smoking cessation. It is important to evaluate if the patient is nicotine-dependent or not, the quantity smoked, desire to quit, presence of associated diseases and feasible treatment modalities^4^^,^^6^.

There are many ways to assess nicotine dependency: through the International Classification of Diseases (ICD-10), Diagnosis and Statistics Manual of the American Psychiatric Association (DSM-IV), and others^4^. The Fagerström Scale for nicotine tolerance and dependency assessment (Table 1) has English and Swedish originals and has been adapted to several languages. It includes six questions. Total score ranges from zero to 11, where low nicotine dependency (mild) is detected when total is below three. A score higher or equal to seven indicates high nicotine dependency (severe)^6^. Patient should be encouraged to quit smoking at each medical consultation. Other smokers in the family must also be counseled not to smoke^3^. According to Jain (2003)^13^, gradual reduction or quit attempts to smoking cessation show the same probability of success.

**Table 1:** Portuguese version of Fagerström scale for nicotine-dependence evaluation

Questão	Resposta	Pontuação
1. Quanto tempo você demora para fumar o primeiro cigarro da manhã?	menos de 5 minutos	3
	6-30 minutos	2
	31–60 minutos	1
	mais de 60 minutos	0
2. É difícil abster-se e não fumar nos lugares onde é proibido (p. ex., hospital, biblioteca, igreja, ônibus, etc.)?	sim	1
	não	0
3. Se tivesse de escolher, que cigarro lhe custaria mais deixar de fumar?	o primeiro da manhã todos os demais	1
		0
4. Quantos cigarros você fuma por dia?	10 ou menos	0
	11–20	1
	21–30	2
	31 ou mais	3
5. Habitualmente você fuma mais nas primeiras horas do dia do que no restante do dia?	sim	1
	não	0
6. Você fuma estando doente na cama?	sim	1
	não	0

NOTE: score from zero to three: low nicotine-dependency (mild); score higher or equal to seven indicates high nicotine-dependency (severe).

#### Pharmacotherapy

Pharmacotherapy is indicated for nicotine dependents and is divided into: first-line therapy (bupropion and nicotine-replacement therapy) and second-line therapy (clonidine and nortriptyline).

### First-line Therapy

#### Bupropion

Bupropion is a non-tricyclic antidepressive that inhibits pre-synaptic dopaminergic and noradrenalin mechanisms^8^^,^^14^^,^^15^. Its action in the central dopaminergic pathways is believed to be the same mechanism responsible for craving reduction in patients under nicotine abstinency^14^.

In the United States, bupropion is indicated for addicts smoking 15 or more cigarettes/day or presenting depressive symptoms^4^.

Bupropion therapy should start 7 to 10 days before patient stops smoking, since this interval is necessary for the balance of pharmacotherapeutic levels^2^^,^^8^. The recommended dosage is 150mg/day up to the third day of treatment, increasing to 300mg/day at the fourth day, and maintaining this dosage from 7 up to 12 weeks^4^.

Clinical studies with bupropion have satisfactory results, presenting twice the period of abstinence when compared with placebo, plus reduced weight gain^4^.

Bupropion's adverse effects occur in 6–8% of patients^16^. The most common symptoms are: insomnia, restlessness and xerostomia 8. Kolber et al. (2003)^16^ emphasize that incidence of adverse effects was observed in clinical studies sponsored by a pharmaceutical manufacturer, in which 35% of patients had not completed treatment.

The authors carried out an independent study to evaluate 39 patients, out of which 15 (38%) discontinued the use of bupropion due to adverse neuropsychiatric effects (trembling, restlessness, and confusion), insomnia and skin eruptions. Seven patients (18%) had to reduce bupropion dosage to 150 mg/day, so side effects could be tolerated.

Risk of convulsions in bupropion users is 1:1.000. For this reason, this drug is contraindicated for epileptics^4^. Other contraindications include: nutrient disorders (nervous anorexia or bulimia), uncontrolled arterial hypertension, recent alcohol abstinence and use of monoaminoxidase inhibitors (tranylcypromine or selegiline)^2^^,^^4^^,^^8^.

Bupropion is a B-category drug according to Food and Drug Administration, which means that there are not sufficient studies on secure use of this medicine during pregnancy^4^.

#### Nicotine Replacement Therapy (NRT)

Combined use of NRT and bupropion almost doubles the success rate of smoking cessation^14^.

In Brazil, nicotine patches and chewing gum are available in the market. In the United States, there is also the nasal spray and nicotine mouthwash^14^.

Patches may be found in the Brazilian market in dosages of 7, 14 and 21mg/unit and each pack contains seven units. They maintain blood levels of nicotine for 16 to 24 hours^17^, therefore they should be replaced on a daily basis. Their effects are observed in two to three days of use^18^. Mean period for treatment is eight weeks^4^.

Chewing gums contain 2mg of nicotine/unit and are sold in packages of 12 units.

The following dosage is recommended^18^^,^^19^:

a) For patients who smoke ≤ 25 cigarettes per day:
  1 gum (2mg) at 1–2 hour intervals in the first 4 weeks up to maximum of 20 gums per day^18^
  1 gum (2mg) at 2–4 hour intervals from the 5th to 8th week
  1 gum (2mg) at 4–8 hour intervals from the 9th to 12th week
b) For patients who smoke > 25 cigarettes per day:
  2 gums (4mg) at 1–2 hour intervals in the first 4 weeks up to maximum of 20 gums per day^18^
  1 gum (2mg) at 2–4 hour intervals from the 5th to 8th week
  1 gum (2mg) at 4–8 hour intervals from the 9th to 12th week

Gums should be strongly chewed until numbness of the mouth mucosa occurs or a tobacco taste is perceived. Then the patient should stop chewing and maintain the gum between the cheeks and gingival region until numbness disappears, and restart chewing for 30 minutes to throw out the gum. Patient should not ingest any type of liquid while chewing the gum^19^.

The patient must stop smoking as soon as he starts NRT. The most common systemic effects in nicotine replacement are: nausea, hiccups and headache18,20. Main adverse effect of nicotine gums is rash of mouth mucosa^4^.

NRT is contraindicated for individuals younger than 18 years and those with severe cardiovascular diseases (acute myocardial infarction occurred within the previous two weeks and instable angina)^4^. Use of NRT is possible in nicotine-dependent pregnant women and during breastfeeding, should treatment risks and benefits be appraised^2^.

#### Second-Line Therapy

Clonidine may be used at a 0.1 to 0.75 mg dosage per day to relieve nicotine-abstinence syndrome's symptoms. Its main adverse effects are sedation and orthostatic hypotension. Sudden discontinuation of clonidine may produce hypertensive crisis^4^.

Nortriptyline inhibits noradrenalin and dopamine mechanisms in the CNS, producing antidepressive and anxiolytic effects. At short-term, its efficacy in smoking cessation seems to be similar to that of bupropion^4^.

### Other therapies

#### Acupuncture

The Acupuncture Consensus Panel of the United States National Institutes of Health (NIH) (1998)^21^ confirms that acupuncture “may be useful as a supportive treatment, or acceptable alternative, or part of a comprehensive program” in drug-addiction therapy, including nicotine dependency. According to Approach Consensus and Treatment of Smokers of the Health Ministry (2001)^19^, “so far, there are not sufficient scientific evidences to corroborate the efficacy of acupuncture and of other methods, such as aromatherapy and hypnosis. Thus, acupuncture “is not recommended as a method of choice for smoking cessation”, although it may be used “if this is the patient's option and if there are no usage contraindications”.

He et al. (2001)^22^ followed 46 adults who smoked 10 or more cigarettes per day and divided them into two groups. The study group was submitted to electroacupuncture, auriculoacupuncture and auriculoacupressure (manual technique, without needles) for three weeks. The activated spots corresponded to the lungs, airways and mouth. Individuals of the control group were submitted to acupuncture with stimulation of spots related to the muscle-skeletal system, presumably without influence in the organs affected by tobacco. Among the study group patients, 32% abandoned smoking, against 23% of the control group. The desire to smoke was reduced in both groups, although tobacco taste significantly worsened among those submitted to acupuncture. According to the authors, acupuncture action mechanisms in smoking treatment remain unknown, although it is possible that tobacco taste is reduced by this technique, with consequent fall of smoking desire.

So far, there are not sufficient evidences that acupuncture is effective in treating nicotine dependency^13^^,^^23^, even though several patients feel better during smoking abstinence^13^.

#### Cognitive-behavioral Therapy and Self-support groups

Marques (2001)^4^ emphasizes that self-support groups and psychotherapy – individual or group – with counseling sessions are effective adjuvant factors in treating nicotine dependency. This is especially significant when dependency is followed by other affections, such as depression and anxiety.

Counseling helps to identify situations in which the tobacco-addicted chases a cigarette due to behavioral (after the meals, a cup of coffee, when meeting friends) or emotional reasons (anxiety, upsetting). Based on that, the tobacco-addicted learns several strategies to break the link between these factors and the act of automatic smoking^14^.

Behavioral intervention and counseling is the base of treatment against tobacco-use among teenagers^2^.

The National Cancer Institute (INCA) has a toll-free phone number 0800-703-7033, where information on smoking cessation methods is provided. In the call center menu options, INCA informs the phone numbers of state coordination centers for smoking treatment under the Central Healthcare System (SUS). Smokers that participate in smoking cessation groups have the right to receive pharmacotherapy without charge.

#### Assessment of Response to Treatment

Routinely, the main information for the physician to evaluate smoking reduction or cessation is self-reported smoking cessation. However, in clinical studies, it is fundamental to adopt an objective and secure measurement to make sure that the patient has really quit smoking.

The most effective method in clinical research studies for smoking cessation is blood, saliva or urine cotinine tests^22^.

Another methodology is measurement of carbon monoxide (CO) in exhaled air. In the respiratory tract CO synthesis occurs through hemoxygenase enzymes, in proportional quantity as to local inflammatory process. Tobacco-addicted usually presents high levels of CO in exhaled air. Approximately 24 hours after smoking cessation, CO exhaled levels start to fall, indicating pulmonary functional recovery. Exhaled CO concentration of non-smokers is below 10 ppm (parts per million)^24^.

Some researchers use portable devices to quantify exhaled CO, not only for focused clinical evaluation, but also to encourage the patient during smoking cessation program enrollment^24^.

#### A National Study

Haggsträm et al. (2001)^20^ assessed 169 smokers which voluntarily enrolled in a university smoking cessation service. Most people who searched for assistance were women (67%), median age (mean 46 years), high educational level and motivated to quit smoking due to respiratory disorders (85%). Nicotine dependency was moderate in 50% of the cases, mild in 27% and severe in 22%. Proposed treatment was cognitive-behavioral psychotherapy for mild cases, psychotherapy associated with pharmacotherapy (or bupropion 300mg/day or NRT) for moderate cases, and psychotherapy associated with pharmacotherapy (bupropion 300mg/day plus NRT) for severe cases. About 30% of smokers abandoned the program in the first week. At the end of the study, 124 individuals remained in the program; 49% had quit smoking and 13% significantly reduced cigarette consumption. Success rate in smoking cessation was: 23% in psychotherapy, 50% in NRT, 59% in bupropion use and 59% in combined use of bupropion and NRT. Only one patient had to interrupt the use of bupropion due to adverse effects.

## DISCUSSION

Ironically, after five centuries, tobacco has gone from a medicinal plant –used even to prevent cancer – to one of the worst world public health issues.

Nicotine dependency is currently one of the most common chronic diseases in the population^24^. Differently from alcohol and illicit drugs, nicotine does not cause acute conditions due to overdose in addicted individuals. Also, it does not lead to aggressive behavior or worsens the psychomotor performance in car driving and machine operation. Therefore, nicotine dependency is less shocking to society than alcohol dependency and other psychoactive drugs. Exceptionally, tobacco addicts are seen as inconvenient or dangerous – although, there is risk to accidentally burn furniture, clothes, tablecloths or mattresses and causing disastrous fire.

On the other hand, an increasing number of people show their disgust to tobacco and to passive smoking. In public places, where smoking is still not prohibited by law, smokers' segregation is commonly seen. These measures protect non-smokers, although they are not effective to solve nicotine dependency.

Governmental campaigns against smoking have been intensified in the last decade, especially through the media and the warnings printed on cigarette packs.

However, around ¼ of the Brazilian population is nicotinedependents and is subjected to the morbimortality caused by tobacco4. Presumably, effective anti-tobacco advertising is useful to make people aware of tobacco negative effects on health, although not sufficient to eliminate nicotine dependency.

Most tobacco addicts are aware of cigarettes harms and want to quit smoking^8^. However, overcoming practical challenges to achieve this goal include: 1) lack of medical diagnosis on nicotine dependency; 2) abstinence discomfort; 3) insufficient number of smoking cessation supportive services and free distribution of medicine by the public health system.

It is difficult to compare scientific studies outcomes related to efficacy of smoking cessation. Several factors must be considered, such as: patients' cultural and socioeconomic features, reasons for their enrollment on smoking cessation program (spontaneous attitude or disease, such as cancer and COPD), nicotine-dependency grade, follow-up period and criteria to assess treatment success rate (objective or subjective).

Invasive treatments due to smoking complications, for instance, have great influence on smoking cessation. Laryngectomized patients due to cancer treatment have a two-fold probability of smoking abstinence than those treated with radiotherapy only^3^.

According to the literature, bupropion is effective in nicotine-dependency treatment, however there are several clinical conditions that contraindicate its use^2^^,^^4^^,^^8^. Presence of side effects is relatively significant, leading to dosage reduction or drug discontinuation in about 38% of the cases^16^.

Nicotine replacement therapy presents good outcomes when associated with bupropion, although it also has limitations, besides not being considered for patients with severe cardiovascular diseases4.

Acupuncture is a controversial method for smoking cessation^23^, especially because western Medicine has not sufficient knowledge on this technique. Scientific occidental literature tends to consider it innocuous for smoking cessation treatment, although, when practiced by a skilled physician, the positive effects of acupuncture cannot be denied. Moreover, effectiveness mechanisms of acupuncture remain unknown and require thorough research.

## CLOSING REMARKS

Otorhinolaryngologists must be aware of available therapeutic modalities for nicotine dependence. Scientific knowledge, in addition to sensitivity and keenness will allow the physician to choose the most adequate and motivating way to encourage patients to quit smoking, reduce unpleasant symptoms of abstinence and avoid relapses.
